# Supplementary material for: The biogeographic differentiation of algal microbiomes in the upper ocean from pole to pole
Source: Nat Commun. 2021 Sep 16;12:5483. doi: 10.1038/s41467-021-25646-9 (PMC8446083; doi:10.1038/s41467-021-25646-9)
Supplement: Supplementary file 1 — Supplementary information [file 41467_2021_25646_MOESM1_ESM.pdf]

Supplementary Information:
The biogeographic differentiation of algal microbiomes in the upper ocean from pole to pole

Kara Martin[1,2&], Katrin Schmidt[3&], Andrew Toseland[3], Chris A. Boulton[4], Kerrie Barry[5], Bánk Beszteri[6], Corina P. D. Brussaard[7], Alicia Clum[5], Chris G. Daum[5], Emiley Eloe-Fadrosh[5], Allison Fong[8], Brian Foster[5], Bryce Foster[5], Michael Ginzburg[8], Marcel Huntemann[5], Natalia N. Ivanova[5], Nikos C. Kyrpides[5], Erika Lindquist[5], Supratim Mukherjee[5] Krishnaveni Palaniappan[5] T.B.K. Reddy[5] Mariam R. Rizkallah[8], Simon Roux[5], Klaas Timmermans[7], Susannah G. Tringe[5], Willem H. van de Poll[9], Neha Varghese[5], Klaus U. Valentin[8], Timothy M. Lenton[4], Igor V. Grigoriev[5], Richard M. Leggett[2], Vincent Moulton[1], Thomas Mock[3*]

[1]School of Computing Sciences, University of East Anglia, Norwich Research Park, NR47TJ, Norwich, United Kingdom
[2]Earlham Institute, Norwich Research Park, Norwich, NR4 7UG, United Kingdom
[3]School of Environmental Sciences, University of East Anglia, Norwich Research Park, NR47TJ, Norwich, United Kingdom
[4]Global Systems Institute, University of Exeter, EX4 4QE, United Kingdom
[5]DOE-Joint Genome Institute, 1 Cyclotron Road, Berkeley, CA 94720, U.S.A
[6]Department of Biology, University of Duisburg-Essen, Essen, Universitaetsstrasse 2, 45141 Essen, Germany.
[7]Royal Netherlands Institute for Sea Research, Texel, The Netherlands
[8]Alfred Wegener Institute for Polar and Marine Research, Am Handelshafen 12, 27570 Bremerhaven, Germany
[9]Department of Ocean Ecosystems, Energy and Sustainability Research Institute Groningen, University of Groningen, Nijenborgh 7, 9747 AG Groningen, The Netherlands

[&]Authors contributed equally

*Corresponding author's email address: t.mock@uea.ac.uk

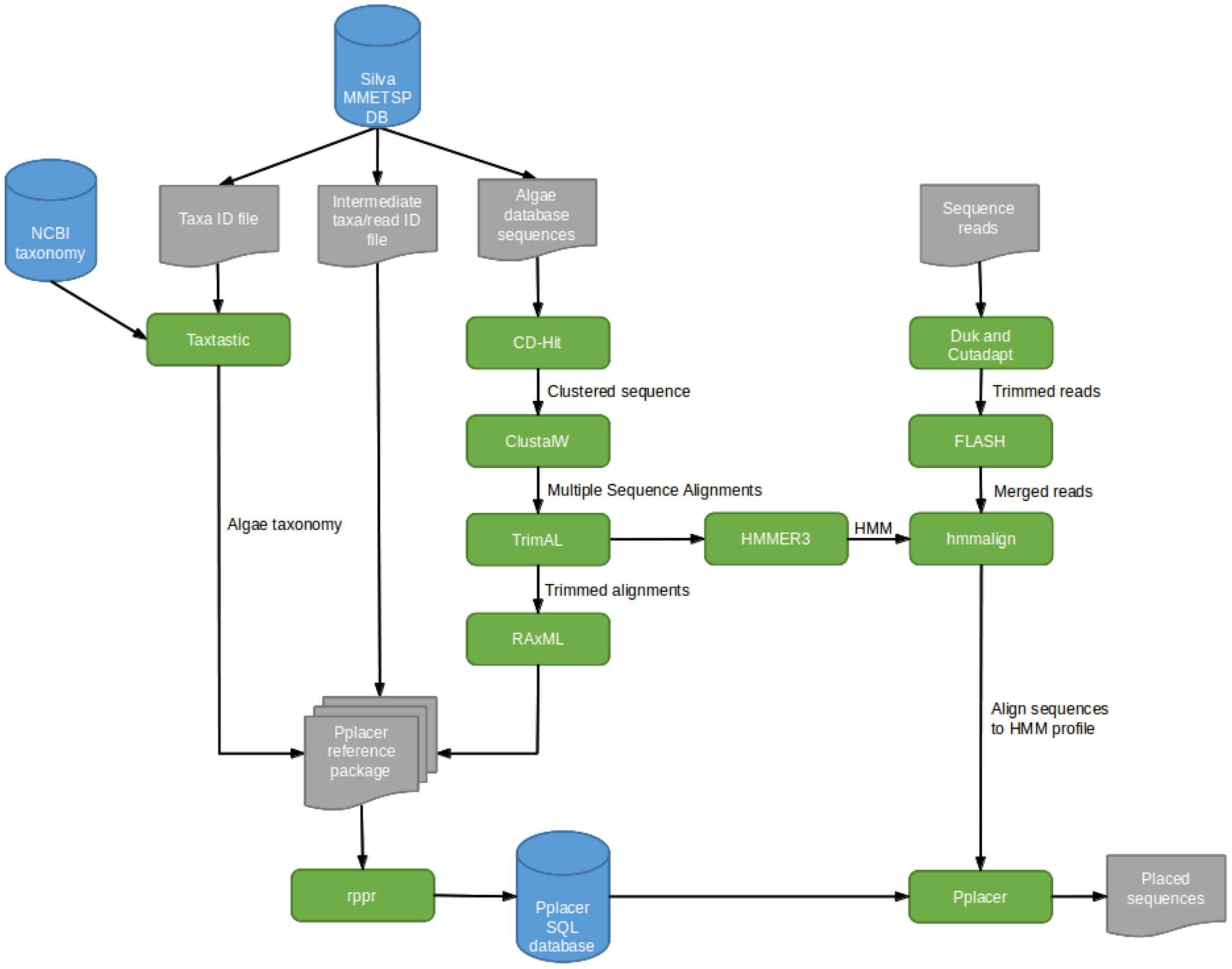

Supplementary Fig. 1. Pipeline. Diagram of Pplacer 18S rDNA classification.

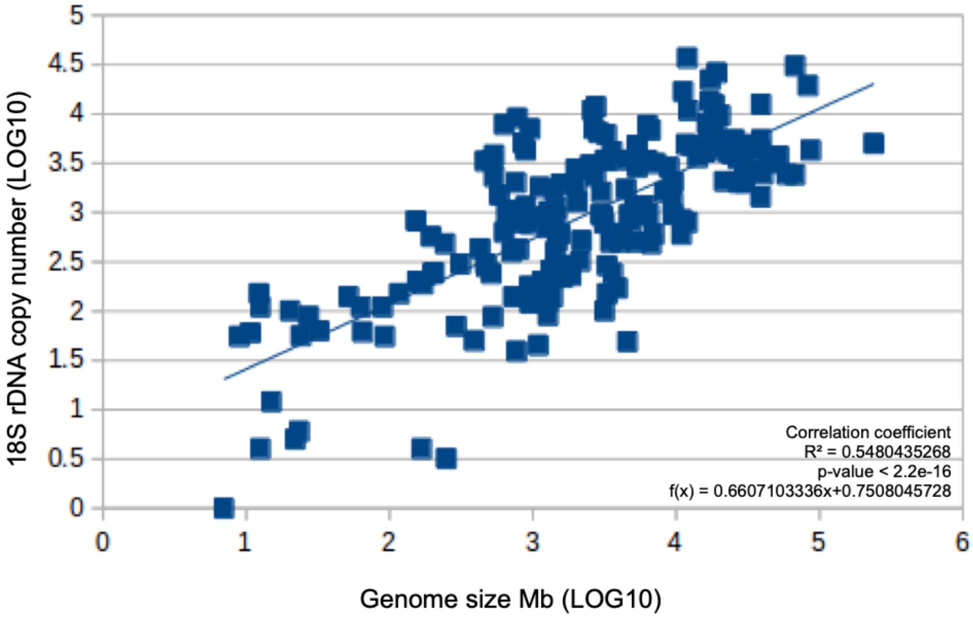

Supplementary Fig. 2. Copy number equation of the line. 18S rDNA copy number and their related genome size (Mb) for 185 species across the eukaryote tree of life (Supplementary Table 4). We investigated 18S rDNA gene copy number and their related genome sizes[1,3,4,5,6,7,8,9,10,11,13]. We observed a significant correlation ($R^2 = 0.55$) with a p-value = 2.2e-16 between genome size and 18S rDNA copy number. Based on the log transformed data a regression equation was determined, f(x)=0.6607103336X+0.7508045728 and used for normalising the 18S rDNA copy number.

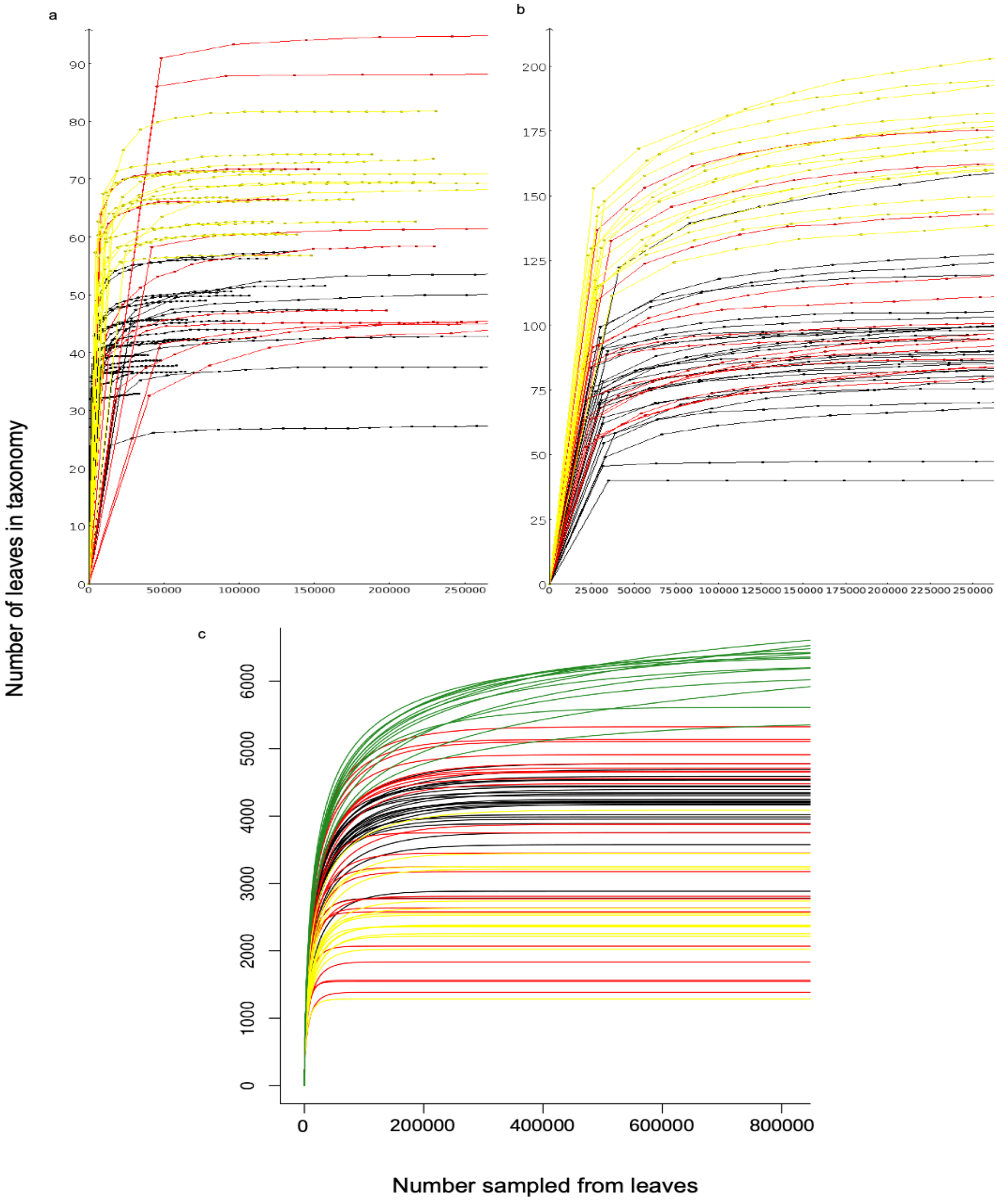

Supplementary Fig. 3. Rarefaction curves. (a) Rarefaction curves of 18S rDNA species level (n=54), **(b)** rarefaction curves of 16S rDNA genus level (*n*=57) and **(c)** rarefaction curves for metatranscriptome Pfam protein families (*n*=79). The *x*-axis represents the random subsample size taken from the dataset and the *y*-axis indicates the number of unique entities found. In each panel the colours correspond to the sample locations of the four expeditions, North Atlantic Ocean in red, Arctic Ocean in black, South Atlantic Ocean in yellow and Antarctic Ocean in green.

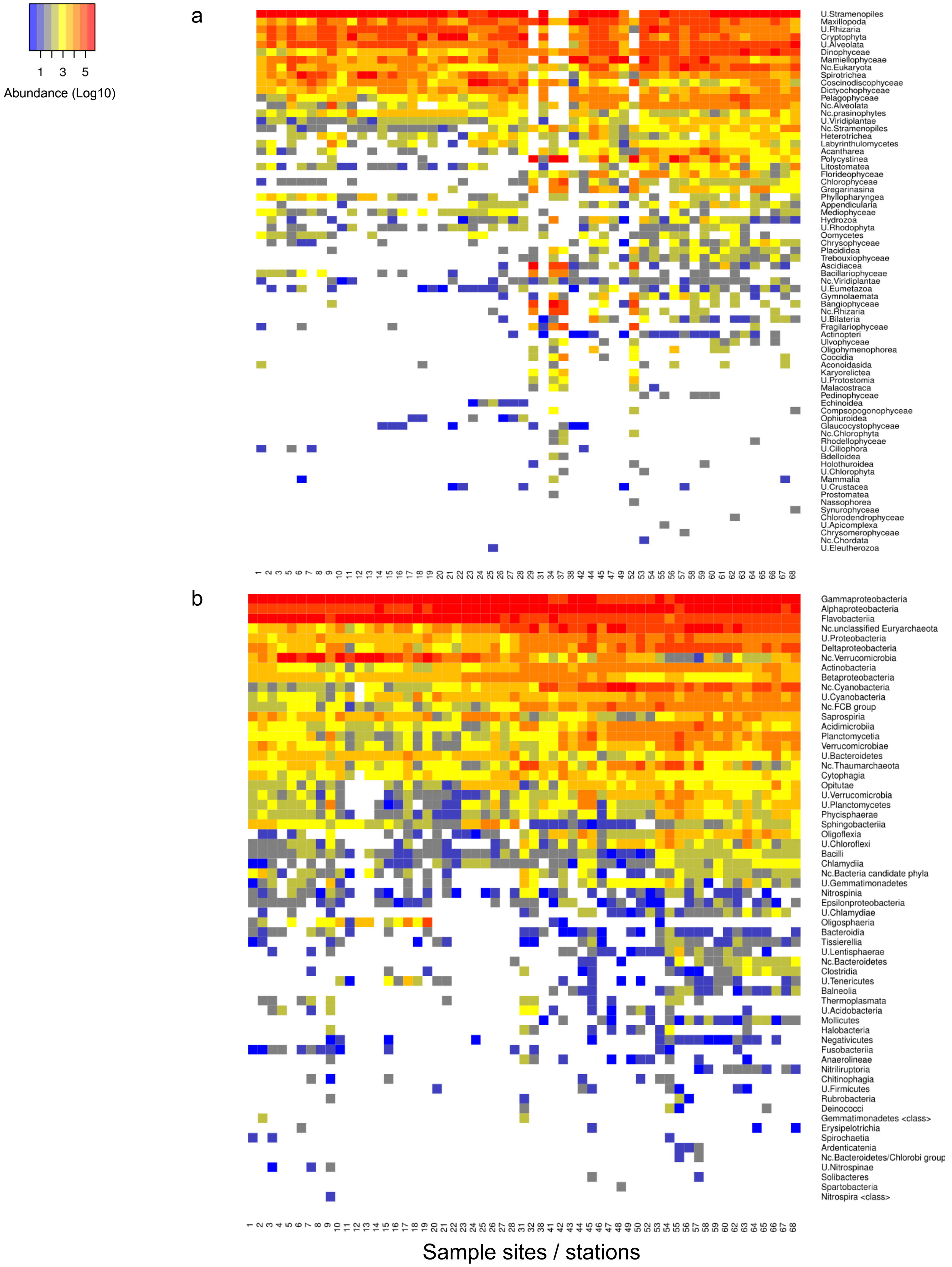

Supplementary Fig. 4. Heatmaps. **(a)** Heatmap of 18S rDNA dataset and **(b)** heatmap of 16S rDNA dataset. The heatmaps are arranged by latitude to the taxonomic rank of class. The numbers correspond to sample locations as shown in Fig. 1a. Colours correspond to the abundance of 18S and 16S rDNA, where red colours are high values and blue colours are low values.

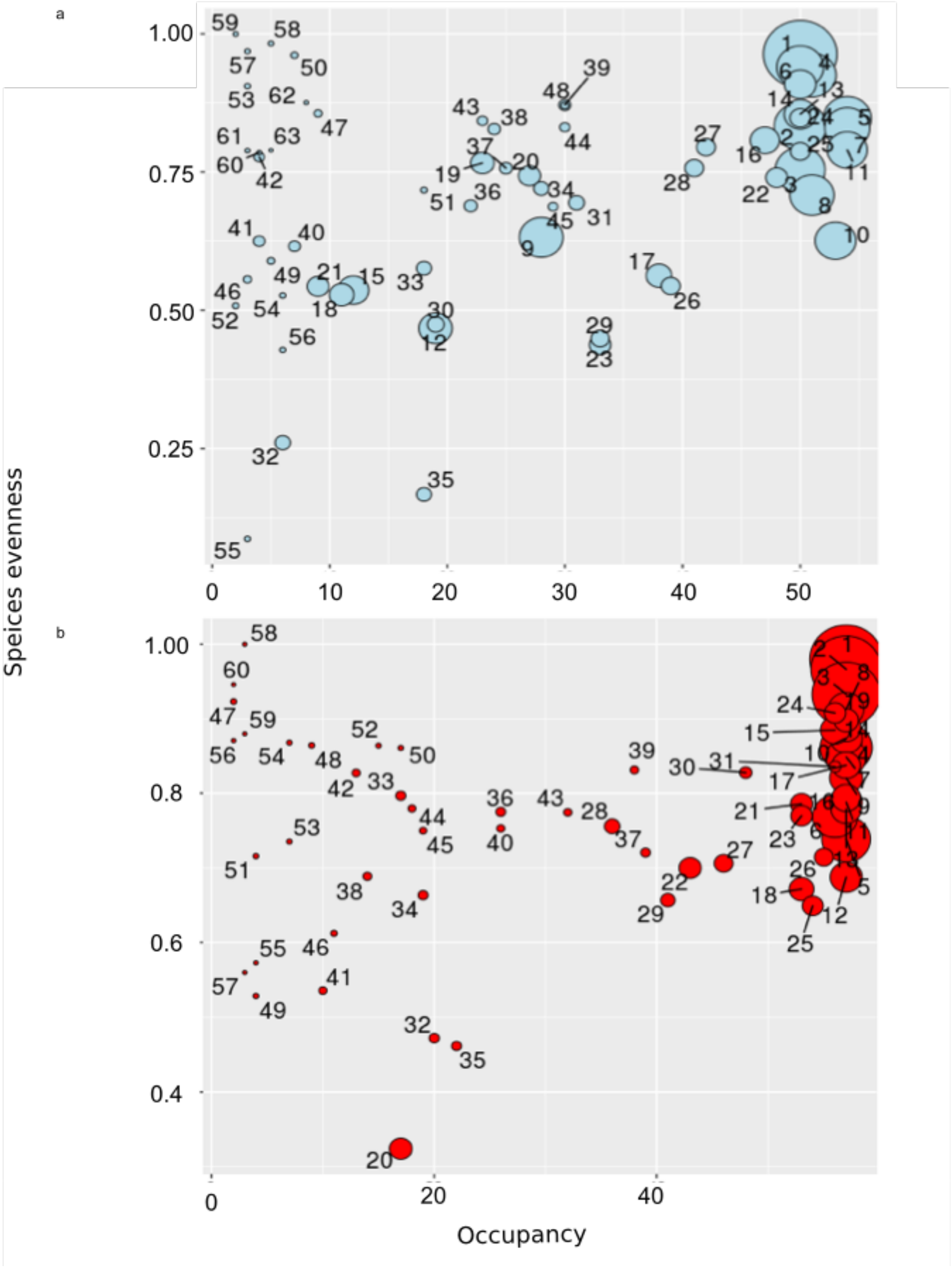

Supplementary Fig. 5. Abundance-based evenness and occupancy plots. (a) and **(b)** represents the 18S and 16S rDNA datasets, respectively. The numbers in the plots correspond to taxon names, which can be found in the Supplementary Table 3. A list of taxa excluded from the analyses due to insufficient data are listed in Supplementary Table 3. The *x*-axis represents the number of times that class taxonomy occurs across the stations. The *y*-axis represents the evenness of that class taxonomy across stations it occurs in. Each circle represents a class taxonomy abundance. The size of each circle corresponds to the total abundance for that class, calculated by taking the square root of the abundance divided by pi.

a

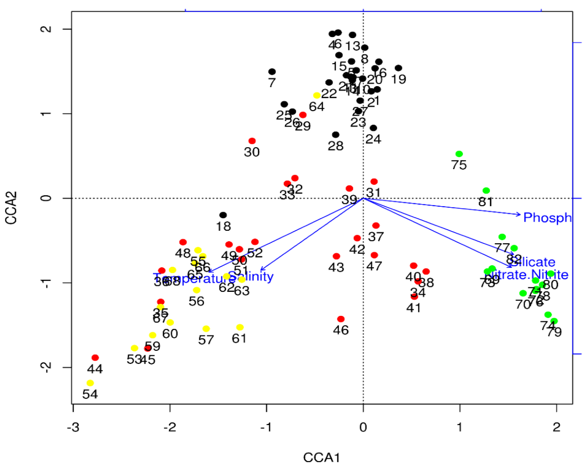

b

	Temperature	Salinity	Nitrate/Nitrite	Phosphate	Silicate
**Metatranscriptome Pfam dataset (n=79):**	0.08025	0.04722	0.07495	0.07785	0.07503
**16S rDNA dataset (n=57):**	0.142	0.08499	0.03084	0.0663	0.05178
**18S rDNA dataset (n=54):**	0.1084	0.0827	NA	0.0544	0.04135

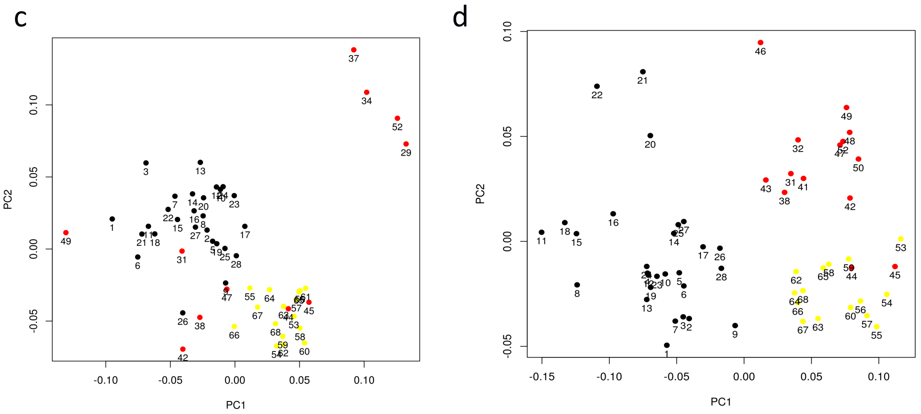

Supplementary Fig. 6. Canonical Correspondence Analysis (CCA) plot and Principal coordinates analysis (PCoA). (a) Canonical Correspondence Analysis (CCA) plot of the metatranscriptome Pfam protein families dataset (*n*=79). The numbers in the plots correspond to sample locations as given in Fig. 1a. The colours correspond to the sample locations of the four expeditions, North Atlantic Ocean in red, Arctic Ocean in black, South Atlantic Ocean in yellow and Antarctic Ocean in green. The arrows represent the direction and the length of the vector. Each vector represents an environmental factor variable. **(b)** represents a table of CCA on each dataset of 18S, 16S rDNA and metatranscript Pfam against the individual environmental variables. The numbers in the table are the percentage of the variation that each variable accounts for in each dataset. **(c)** and **(d)** represents coordinates analysis (PCoA) of communities at all leaves at the taxonomic rank of class. **(c)** represents the eukaryotic classes (n=54) and **(d)**, represents the prokaryotic classes (n=50). The communities are clustered according to their similarity based on Bray-Curtis distances. The numbers correspond to sample locations as given in Fig. 1a. The yellow dots are the samples collected during November to December of 2012. These samples span the South Atlantic Ocean. The red dots are the Stratiphyt samples, which span from the Canaries to Iceland. These samples were collected during April to May of 2011. In black dots are samples collected during June to July of 2012. These are the Arctic Ocean samples spanning the west Spitsbergen current, east Greenland current and Norwegian Atlantic current.

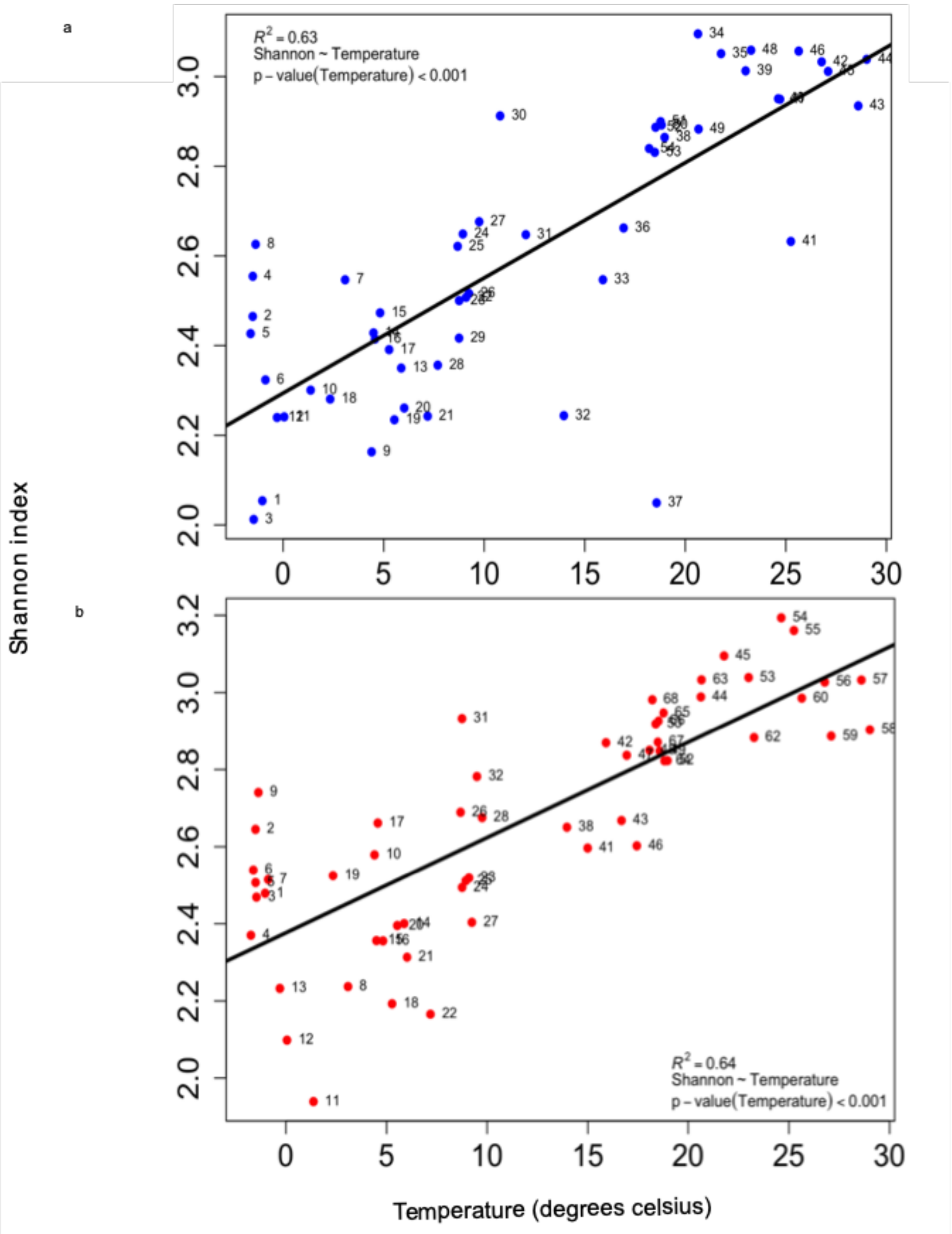

Supplementary Fig. 7. Regression analysis. (a) a positive correlation of 18S rDNA diversity based on Shannon index with temperature. Based on backward model selection, temperature was the only significant environmental covariate determined. **(b)** a positive correlation of 16S rDNA diversity, based on Shannon index with temperature. Based on backward model selection, temperature was the only significant environmental covariate determined. The numbers correspond to sample locations as shown in Fig. 1a.

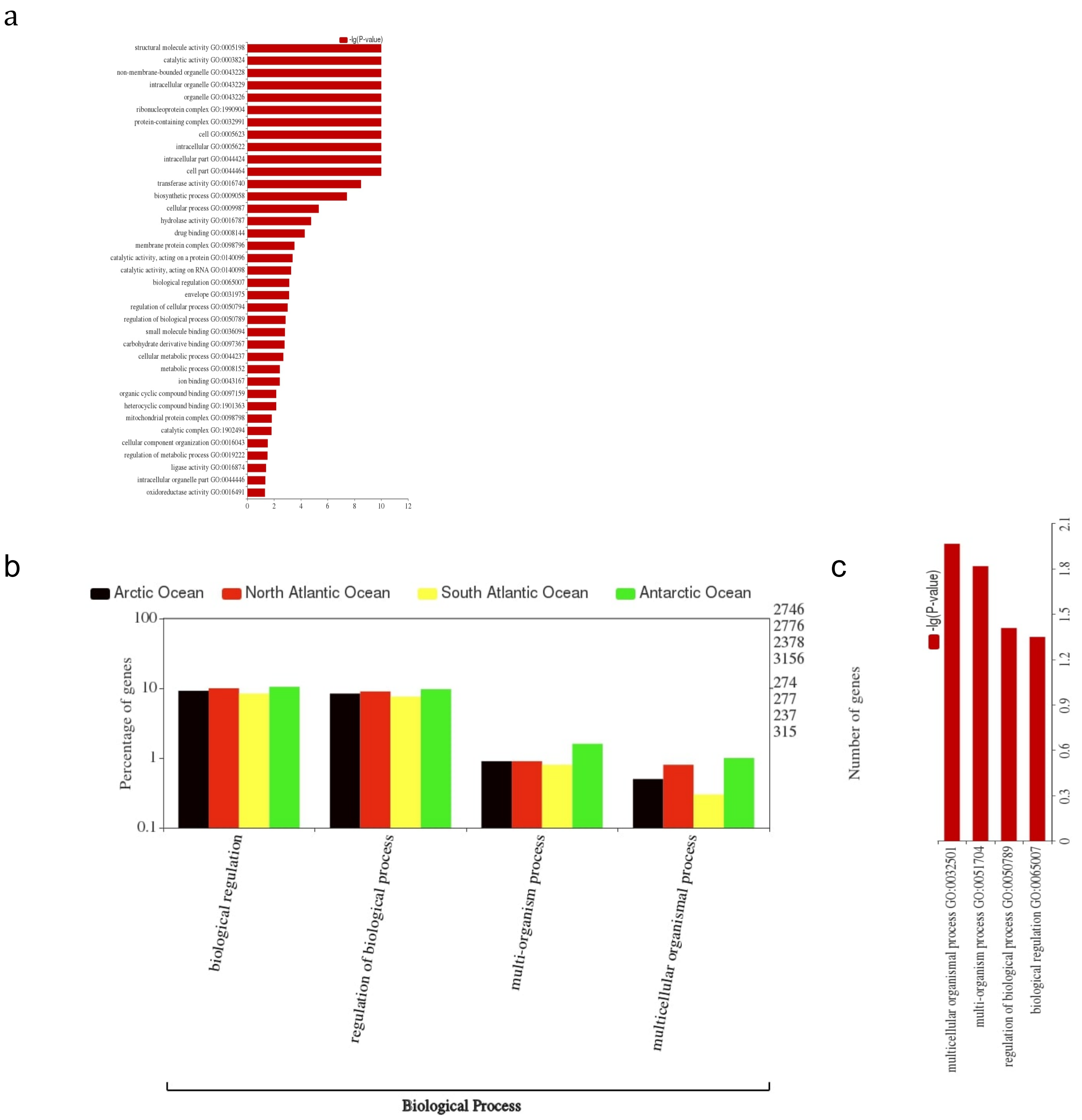

Supplementary Fig. 8. Gene Ontology (GO) analysis. Gene Ontology (GO) analysis of the co-occurrence of Pfam protein families dataset (*n*=79), in which two networks were found, a cold network (*n*=2369) and warm network (*n*=1614). These each were mapped to respective GO terms and plotted. Displayed in panel (**a**) is the p-values for the GO terms analysis in Fig. 2c. **(b)** GO terms analysis for the Pfam protein families dataset (*n*=79). Each Pfam was mapped to respective GO terms and plotted. The colours correspond to the sample locations of the four expeditions, North Atlantic Ocean in red, Arctic Ocean in black, South Atlantic Ocean in yellow and Antarctic Ocean in green. Displayed in panel **(c)** is the p-values for the GO terms analysis in panel **(b).** (Figures was generated with http://wego.genomics.org.cn/).

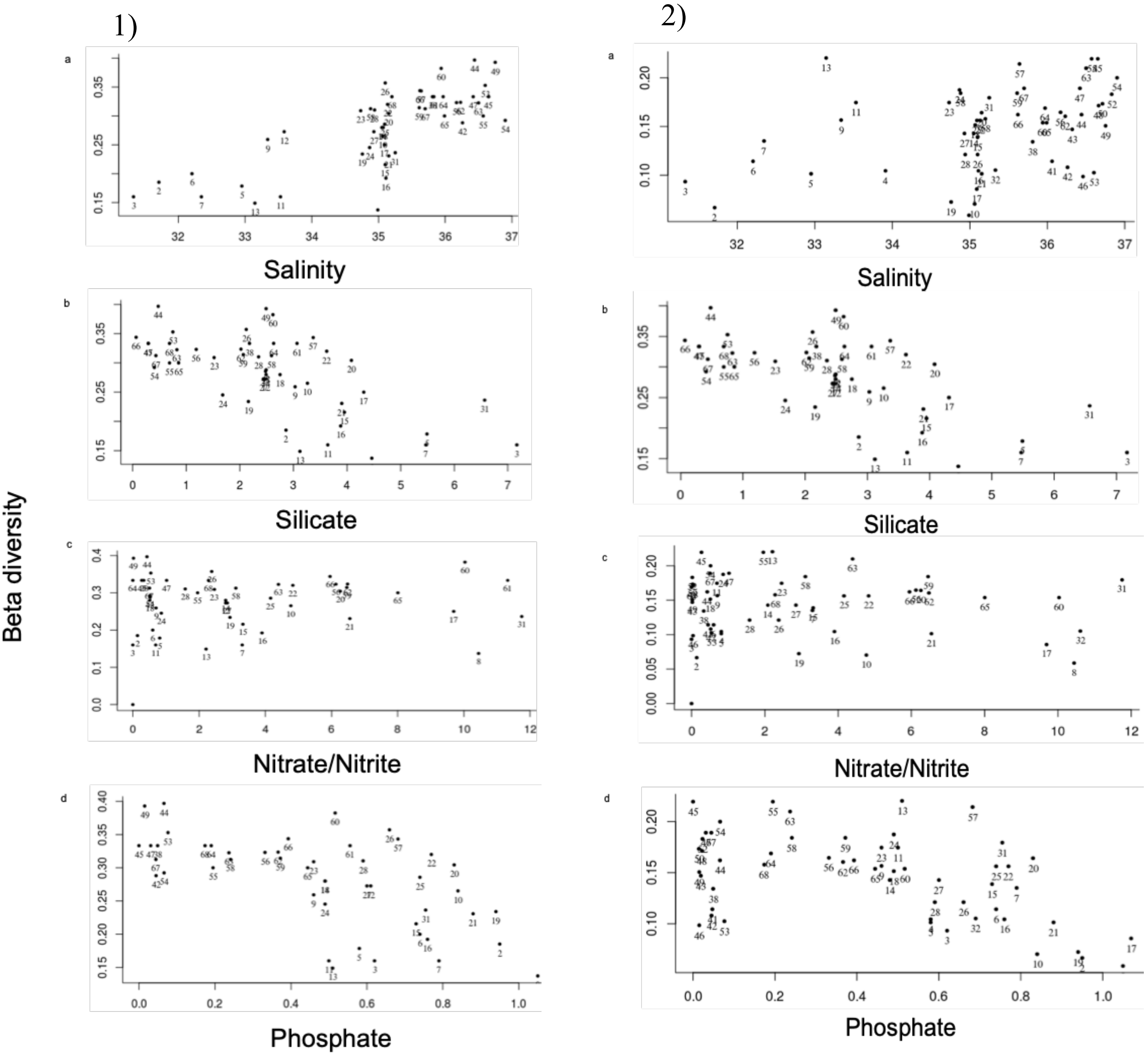

Supplementary Fig. 9. Plot of beta diversity against environmental variables. (1) 18S rDNA and **(2)** 16S rDNA dataset of beta diversity plotted against the environmental variables. The numbers correspond to sample locations as shown in Fig. 1a. The *y*-axis represents the beta diversity across the stations. The *x*-axis in panels (**a**), **(b)**, **(c)** and **(d)** represents salinity, silicate, nitrate/nitrite and phosphate. Nutrient concentrations are given in μmol L^{-1}, and the unit for salinity is PSU (Practical Salinity Unit).

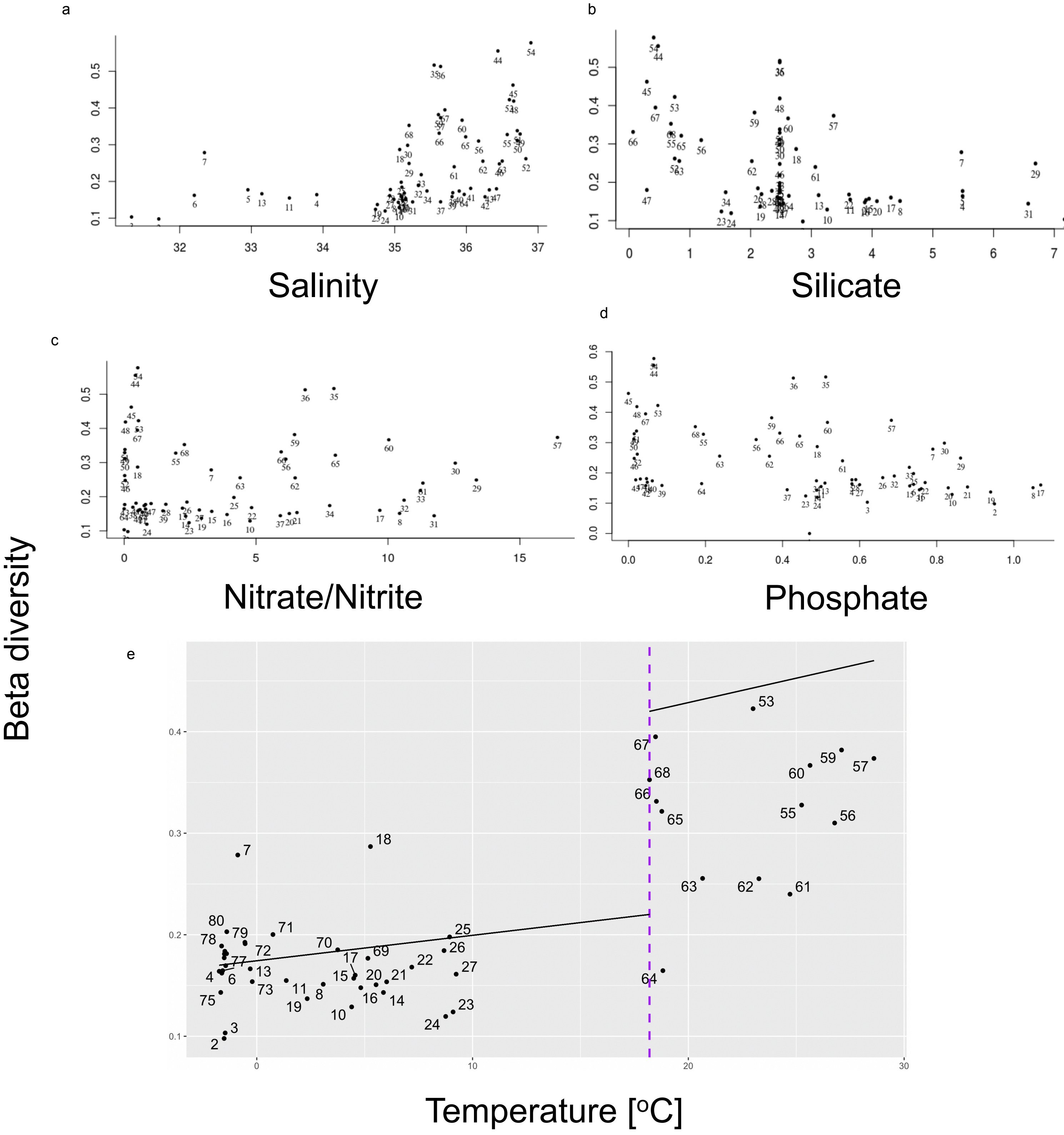

Supplementary Fig. 10. Breakpoint analysis of beta diversity against environmental variables. Pfam protein families dataset (*n*=79) beta diversity plotted against environmental variables. The numbers correspond to sample locations as shown in Fig. 1a. The *y*-axis represents the beta diversity across the stations. The *x*-axis in panels **(a)**, **(b)**, **(c)**, **(d)** and **(e)** represents salinity, silicate, nitrate/nitrite, phosphate and temperature. The breakpoint analysis in panel (**e**) has been done without the North Atlantic data (Stratiphyt-II) to provide a sensitivity test. This analysis resulted in a breakpoint at 18.2°C with a p-value of 1.651e-11, confirming the breakpoint shown in figure 5a, which includes the North Atlantic metatranscriptome data. Nutrient concentrations are given in µmol L^{-1}, and the unit for salinity is PSU (Practical Salinity Unit).

Supplementary Table 1								
Station	Longitude	Latitude	Depth meter	Temperature degrees celsius	Salinity	Nitrate.Nitrite	Phosphate	Silicate
1	-9.52472	79.0225	17	-1.0337	31.0274	0	0.47	2.48
2	-8.52472	79.07611	26	-1.5122	31.708	0.14	0.95	2.86
3	-7.67278	79.04278	20	-1.4645	31.3282	0	0.62	7.17
4	-4.08556	79.00056	35	-1.7398	33.9123	0.81	0.58	5.49
5	-4.08556	79.00056	10	-1.5083	32.9517	0.81	0.58	5.49
6	-4.78556	78.85611	20	-1.6191	32.2039	0.6	0.74	8.97
7	-3.22861	78.86694	15	-0.8805	32.3454	3.3	0.79	5.47
8	-2.83722	78.89667	110	3.0802	34.9887	10.44	1.05	4.46
9	-2.83722	78.89667	25	-1.3686	33.3389	0.7	0.46	3.03
10	-1.84	78.93667	16	4.3973	35.062	4.77	0.84	3.26
11	-0.55917	78.98167	10	1.3665	33.5306	0.69	0.5	3.64
12	3.73583	79.07278	10	0.0522	33.586	2.8	0.61	2.49
13	3.73583	79.07278	5	-0.3027	33.1465	2.21	0.51	3.12
14	4.1527	78.62153	15	5.87	35.05	NA	NA	NA
15	5.32861	78.83889	15	4.4939	35.0994	3.32	0.73	3.95
16	6.10556	79.08944	7	4.8199	35.1105	3.9	0.76	3.88
17	7.07639	79.06667	18	4.5614	35.0886	9.69	1.07	4.31
18	8.11222	78.86972	10	5.269	35.0693	0.51	0.49	2.75
19	9.23306	78.85139	25	2.3374	34.7579	2.93	0.94	2.16
20	11.30917	76.25389	15	5.5282	35.1514	6.26	0.83	4.08
21	9.85667	73.01889	20	6.0186	35.1528	6.55	0.88	3.9
22	8.86667	71.20083	10	7.1834	35.1344	4.83	0.77	3.62
23	7.73028	69.23028	10	9.0976	34.7321	2.46	0.46	1.52
24	7.73028	69.23028	5	8.7545	34.8661	0.86	0.49	1.68
25	6.53028	67.23028	15	8.9384	35.091	4.16	0.74	2.48
26	6.53028	67.23028	20	8.6781	35.1	2.38	0.66	2.12
27	5.41917	65.24611	20	9.24	34.93	2.85	0.6	2.44
28	5.41917	65.24611	5	9.75	34.94	1.58	0.59	2.35
29	-21.7407	62.8	10	7.69	35.2	13.35	0.862	6.69
30	-20.4903	61.7103	11	8.27	35.18	12.55	0.82	NA
31	-19.3398	60.6801	10	8.75	35.25	11.75	0.755	6.57
32	-18.0699	59.5	10	9.49	35.33	10.61	0.69	NA
33	-16.5201	58.0002	11	9.54	35.37	11.23	0.729	NA
34	-16.509	54.6333	10	10.79	35.45	7.79	0.488	1.59
35	-16.4972	52.6218	26	11.54	35.55	7.95	0.512	NA
36	-16.3567	49.9151	50	12.22	35.64	6.86	0.428	NA
37	-12.1104	47.5701	40	12.07	35.64	5.92	0.412	2.54
38	-12.4301	45.5298	16	13.96	35.81	0.33	0.049	2.18
39	-12.6098	44.2798	20	13.78	35.8	1.47	0.087	NA
40	-12.8799	42.3401	21	14.4	35.9	0.76	0.062	NA
41	-13.1901	40.5296	25	14.99	36.06	0.45	0.047	NA

42	-13.576	38.4205	67	15.9	36.26	0.52	0.045	NA
43	-13.94	36.5297	25	16.67	36.32	0.02	0.019	NA
44	-12.0872	37.833	80	20.63	36.44	0.426330313	0.06572038	0.4781875
45	-13.1352	34.876	80	21.78	36.65	0.269427308	0	0.2926839
46	-14.2597	34.7198	21	17.43	36.46	0.04	0.015	NA
47	-14.2601	34.7197	48	16.93	36.42	1.02	0.031	0.2926
48	-14.5898	32.8199	73	18.06	36.66	0.05	0.022	NA
49	-14.8699	31.2202	75	18.57	36.75	0.02	0.015	NA
50	-14.8704	31.2193	72	18.37	36.71	0.03	0.014	NA
51	-15.0705	30.0186	30	18.55	36.71	0.03	0.021	NA
52	-15	29	81	18.97	36.83	0.02	0.023	0.75105
53	-15.1548	28.937	90	23	36.6	0.543261784	0.07633405	0.7510578
54	-17.4585	26.049	80	24.62	36.9	0.516096977	0.06603304	0.4051161
55	-20.1823	23.69	60	25.25	36.57	1.954317104	0.19463208	0.6894914
56	-20.7015	18.755	45	26.78	36.17	6.127505044	0.33145415	1.1872838
57	-20.515	15.249	55	28.6	35.64	16.42369279	0.68233967	3.3670168
58	-18.6202	8.472	46	29.02	34.88	3.106489549	0.24104374	2.5905272
59	-13.602	2.405	80	27.1	35.61	6.458482733	0.3717252	2.0648533
60	-9.4218	-2.045	63	25.64	35.94	10.02985752	0.51653754	2.6174161
61	-7.0612	-4.668	47	24.71	35.83	11.32366081	0.55562155	3.0660004
62	-4.9043	-7.394	45	23.27	36.23	6.480985475	0.36603452	2.0204297
63	-0.3178	-13.104	75	20.66	36.5	4.395764083	0.23672794	0.8268839
64	2.9768	-17.283	30	18.82	35.97	0	0.19	2.63
65	3.7993	-18.25	43	18.77	35.99	8.005879218	0.44437273	0.8590491
66	5.9978	-20.987	30	18.52	35.62	5.951945303	0.39273967	0.0657757
67	7.1922	-22.644	40	18.48	35.7	0.502052705	0.04454304	0.4340192
68	9.9828	-26.441	30	18.2	35.2	2.283057742	0.17364499	0.6887285
69	6.286967	-45.953933	35	5.15	33.84	21.25	1.37	3.1
70	2.8377	-49.012917	50	3.75	33.88	21.93	1.43	3.19
71	2.099283	-51.992633	50	0.75	33.73	25.97	1.7	27.49
72	1.056017	-55.877767	24	-0.54	33.9	21.57	1.46	57.48
73	0.077933	-59.046683	25	-0.21	33.85	24.85	1.76	62.93
74	-0.0007	-60.99955	35	-1.48	34.22	26.93	1.75	63.6
75	-51.595583	-63.482717	40	-1.67	34.2	27.5	1.81	57.12
76	0.06405	-63.998933	35	-1.4	34.28	25.83	1.78	61.02
77	-12.286717	-66.003133	40	-1.58	34.35	29.09	2.02	68.5
78	-0.010367	-66.537983	40	-1.63	34.36	28.26	1.99	74.41
79	-0.0106	-68.012033	33	-0.55	34.35	29	2.15	82.49
80	-0.053333	-69.404583	15	-1.39	34.14	29.76	2.11	64.66
81	-8.28215	-70.365783	30	-1.44	33.76	26.09	2.67	57.49
82	-13.979017	-71.363917	30	-1.5	33.4	27.8	1.94	57.45

Supplementary Table 2			
a			
Cold network:			
Node number	Species	Class	No. connections
1	Colwellia	Gammaproteobacteria	46
2	Polaribacter	Flavobacteriia	46
3	Balneatrix	Gammaproteobacteria	45
4	Ulvibacter	Flavobacteriia	45
5	Amylibacter	Alphaproteobacteria	44
6	Lentibacter	Alphaproteobacteria	44
7	Favella azorica	Spirotrichea	43
8	Phaeocystis cordata	Haptophyceae	41
9	Lentimonas	Verrucomicrobia	40
10	Sulfitobacter	Alphaproteobacteria	40
11	Oceanicoccus	Gammaproteobacteria	39
12	Formosa	Flavobacteriia	38
13	Haliea	Gammaproteobacteria	35
14	Actinocyclus actinochilus	Coscinodiscophyceae	34
15	Chroomonas cf. mesostigmatica	Cryptophyta	34
16	Arenicella	Gammaproteobacteria	33
17	Emcibacter	Alphaproteobacteria	33
18	Ilumatobacter	Acidimicrobiia	32
19	Pyramimonas disomata	Chlorophyta	32
20	Aquibacter	Flavobacteriia	31
21	Glaciecola	Gammaproteobacteria	31
22	Pseudofulvibacter	Flavobacteriia	30
23	Pithites vorax	Phyllopharyngea	27
24	Dokdonia	Flavobacteriia	26
25	Mantoniella antarctica	Chlorophyta	26
26	Mesoflavibacter	Flavobacteriia	26
27	Porticoccus	Gammaproteobacteria	26
28	Sinobacterium	Gammaproteobacteria	25
29	Schizochytrium aggregatum	Labyrinthulomycetes	24
30	Dictyocha fibula	Dictyochophyceae	22
31	Flavicella	Flavobacteriia	21
32	Oleispira	Gammaproteobacteria	21
33	Pseudochattonella verruculosa	Dictyochophyceae	21
34	Pterosperma cristatum	Chlorophyta	21
35	Prasinoderma coloniale	Chlorophyta	20
36	Pseudoscourfieldia marina	Chlorophyta	20
37	Developayella elegans	Stramenopiles	19
38	Ellobiopsis chattonii	Alveolata	19
39	Pseudohongiella	Gammaproteobacteria	19
40	Francisella	Gammaproteobacteria	18
41	Neptuniibacter	Gammaproteobacteria	18
42	Marivita	Alphaproteobacteria	16
43	Paraglaciecola	Gammaproteobacteria	16
44	Aureococcus anophagefferens	Pelagophyceae	15
45	Pelagostrobilidium neptuni	Spirotrichea	15
46	Magnetospira	Alphaproteobacteria	14
47	Peritromus kahli	Heterotrichea	12

48	Ceratium tenue	Dinophyceae	11
49	Chrysochromulina parva	Haptophyceae	11
50	Varistrombidium kielum	Spirotrichea	10
51	Florenciella parvula	Dictyochophyceae	7
b			
Warm network:			
Node number	Species	Class	No. connections
1	Erythrobacter	Alphaproteobacteria	62
2	Alteromonas	Gammaproteobacteria	60
3	Roseovarius	Alphaproteobacteria	60
4	Marinobacter	Gammaproteobacteria	59
5	Pelagomonas calceolata	Pelagophyceae	59
6	Prochlorococcus	Cyanobacteria	59
7	Pseudomonas	Gammaproteobacteria	59
8	Synechococcus	Cyanobacteria	59
9	Pelagibaca	Alphaproteobacteria	58
10	Staphylococcus	Bacilli	58
11	Candidatus Actinomarina	Actinobacteria	57
12	Alcanivorax	Gammaproteobacteria	56
13	Croceibacter	Flavobacteriia	56
14	Halomonas	Gammaproteobacteria	56
15	Loktanella	Alphaproteobacteria	56
16	Rhodococcus	Actinobacteria	55
17	Sphingorhabdus	Alphaproteobacteria	55
18	Streptococcus	Bacilli	55
19	Coxiella	Gammaproteobacteria	54
20	Hyphomonas	Alphaproteobacteria	54
21	Lawsonella	Actinobacteria	54
22	Roseibacillus	Verrucomicrobiae	54
23	Candidatus Fritschea	Chlamydiia	53
24	Psychrobacter	Gammaproteobacteria	53
25	Rhodopirellula	Planctomycetia	53
26	Tenacibaculum	Flavobacteriia	51
27	Ichthyodinium chabelardi	Alveolata	50
28	Acinetobacter	Gammaproteobacteria	48
29	Hoeflea	Alphaproteobacteria	47
30	Candidatus Nitrosopelagicus	Thaumarchaeota	46
31	Codonellopsis americana	Spirotrichea	46
32	Euduboscquella crenulata	Dinophyceae	46
33	Halobacteriovorax	Oligoflexia	45
34	Pseudoalteromonas	Gammaproteobacteria	43
35	Idiomarina	Gammaproteobacteria	42
36	Jejudonia	Flavobacteriia	42
37	Blastopirellula	Planctomycetia	41
38	Maricaulis	Alphaproteobacteria	41
39	Pelagococcus subviridis	Pelagophyceae	41
40	Maribacter	Flavobacteriia	39
41	Caecitellus parvulus	Stramenopiles	38
42	Litoricola	Gammaproteobacteria	37
43	Aureispira	Saprospiria	36

44	Filamoeba nolandi	Amoebozoa	36
45	Marinomonas	Gammaproteobacteria	36
46	Thiothrix	Gammaproteobacteria	36
47	Sphingomonas	Alphaproteobacteria	34
48	Oleiphilus	Gammaproteobacteria	33
49	Pseudophaeobacter	Alphaproteobacteria	33
50	Marinoscillum	Cytophagia	32
51	Neptunomonas	Gammaproteobacteria	31
52	Fluviicola	Flavobacteriia	30
53	Hydra vulgaris	Hydrozoa	29
54	Oleibacter	Gammaproteobacteria	29
55	Parabirojimia similis	Spirotrichea	28
56	Olleya	Flavobacteriia	27
57	Bathycoccus prasinos	Mamiellophyceae	21
58	Candidatus Pelagibacter	Alphaproteobacteria	20
59	Kordia	Flavobacteriia	20
60	Nitrosopumilus	Thaumarchaeota	17
61	Nonlabens	Flavobacteriia	10
62	Psychroflexus	Flavobacteriia	10
63	Arcobacter	Epsilonproteobacteria	5
64	Delftia	Betaproteobacteria	5
65	Marinicella	Gammaproteobacteria	5
66	Bradyrhizobium	Alphaproteobacteria	4
67	Rubritalea	Verrucomicrobiae	4
68	Bacillus	Bacilli	3
69	Salinirepens	Flavobacteriia	2
70	Crocinitomix	Flavobacteriia	1

Supplementary Table 3			
Evenness and occupancy plot names to numbers			
a		b	
Number	18S rDNA taxonomy	Number	16S rDNA taxonomy
1	U.Stramenopiles	1	Gammaproteobacteria
2	Cryptophyta	2	Alphaproteobacteria
3	Mamiellophyceae	3	Flavobacteriia
4	U.Rhizaria	4	Nc.unclassified Euryarchaeota
5	Maxillopoda	5	Nc.Verrucomicrobia
6	U.Alveolata	6	Nc.Cyanobacteria
7	Dinophyceae	7	Deltaproteobacteria
8	Spirotrichea	8	U.Proteobacteria
9	Polycystinea	9	Nc.FCB group
10	Coscinodiscophyceae	10	Actinobacteria
11	Nc.Eukaryota	11	Planctomycetia
12	Ascidiacea	12	Nc.Thaumarchaeota
13	Pelagophyceae	13	Acidimicrobiia
14	Dictyochophyceae	14	Betaproteobacteria
15	Bangiophyceae	15	U.Cyanobacteria
16	Nc.Alveolata	16	Verrucomicrobiae
17	Acantharea	17	Saprospiria
18	Nc.Rhizaria	18	U.Verrucomicrobia
19	Gregarinasina	19	U.Bacteroidetes
20	Florideophyceae	20	Oligosphaeria
21	Fragilariophyceae	21	Opitutae
22	Nc.Stramenopiles	22	Oligoflexia
23	Chlorophyceae	23	U.Planctomycetes
24	Nc.prasinophytes	24	Cytophagia
25	U.Viridiplantae	25	Sphingobacteriia
26	Litostomatea	26	Phycisphaerae
27	Heterotrichea	27	U.Chloroflexi
28	Labyrinthulomycetes	28	U.Gemmatimonadetes
29	Hydrozoa	29	Nc.Bacteria candidate phyla
30	Bacillariophyceae	30	Chlamydiia
31	Phyllopharyngea	31	Bacilli
32	Coccidia	32	U.Tenericutes
33	Gymnolaemata	33	Nc.Bacteroidetes
34	Appendicularia	34	Clostridia
35	U.Bilateria	35	U.Lentisphaerae
36	Placididea	36	U.Chlamydiae
37	Chrysophyceae	37	Nitrospinia
38	Oomycetes	38	U.Acidobacteria
39	Mediophyceae	39	Epsilonproteobacteria
40	Oligohymenophorea	40	Tissierellia
41	Karyorelictea	41	Halobacteria
42	U.Protostomia	42	Thermoplasmata
43	Trebouxiophyceae	43	Bacteroidia
44	U.Rhodophyta	44	Balneolia
45	U.Eumetazoa	45	Mollicutes
46	Compsopogonophyceae	46	Anaerolineae

47	Ulvophyceae	47	Gemmatimonadetes <class>
48	Nc.Viridiplantae	48	Nitriliruptoria
49	Malacostraca	49	Deinococci
50	Aconoidasida	50	Negativicutes
51	Actinopteri	51	Rubrobacteria
52	Bdelloidea	52	Fusobacteriia
53	Nc.Chlorophyta	53	U.Firmicutes
54	Echinoidea	54	Chitinophagia
55	Mammalia	55	Erysipelotrichia
56	Ophiuroidea	56	Nc.Bacteroidetes/Chlorobi group
57	Rhodellophyceae	57	U.Nitrospinae
58	Pedinophyceae	58	Spirochaetia
59	U.Chlorophyta	59	Ardenticatenia
60	U.Ciliophora	60	Solibacteres
61	Holothuroidea		
62	Glaucocystophyceae	16S rDNA taxa removed due to insufficient data:	
63	U.Crustacea	Spartobacteria	
		Nitrospira <class>	
18S rDNA taxa removed due to insufficient data :			
Prostomatea			
Nassophorea			
Synurophyceae			
Chlorodendrophyceae			
U.Apicomplexa			
Chrysomerophyceae			
Nc.Chordata			
U.Eleutherozoa			

Supplementary Table 4		
Species_name	Genome_size_Mb	Copy_number
Sabethes_cyaneus	773	39
Haemagogus_equinus	1095	45
Gadus_morhua	391	50
Caenorhabditis_elegans_	93	55
Chironomus_tentans_	293	70
Culex_pipiens_quinquefasciatus	528	87
Temora_longicornis	1281	90
Mus_musculus_	3176	100
Tinca_tinca	959	120
Abramis_brama	1280	125
Pseudopleuronectes_americanus_	734	140
Leuciscus_cephalus_	1381	140
Chlamydomonas_reinhardii_	117	150
Rutilus_rutilus	1117	150
Homo_sapiens_	3319	150
Acheta_domestica	3912	170
Aedes_seatoi	949	178
Drosophila_melanogaster_	172	188
Gallus_domesticus_	1277	192
Drosophila_simulans	156	200
Spisula_solidissima	1167	200
Mytilus_edulis	1619	220
Carassius_auratus_	1848	230
Drosophila_hydei	200	240
Bombyx_mori	513	240
Cricetulus_griseus_	3628	240
Tetrahymena_pyriformis	205	245
Barbus_barbus	1741	250
Lytechinus_variegatus	1340	260
Panagrellus_silusiae	474	280
Rattus_norvegicus	3309	288
Cyprinus_carpio_	1746	290
Ascaris_lumbricoides	313	300
Clupea_harengus	469	301
Artemia_salina	2156	320
Aedes_polynesiensis	714	398
Mesocyclops_edax	1438	400
Aedes_aegypti	799	423
Oryza_sativa_	430	430
Anopheles_quadrimaculatus	245	479
Squalus_acanthias	6735	480
Xenopus_borealis	3487	500
Hyla_cinerea	4046	500
Bufo_viridus	5302	500
Aedes_triseriatus	1487	512
Coregonus_fera	2201	518
Arabidopsis_thaliana_	196	570
Rana_catesbeiana	7025	600

Tetrahymena_thermophila	10934	600
Thymallus_thymallus	1555	608
Bufo_marinus	4222	615
Citrus_sinensis	636	628
Thalictrum_aquilegiifolium	1271	700
Aedes_alcasidi	949	762
Xenopus_laevis	3156	778
Wyeomyia_smithii	841	783
Helianthus_tuberosus	12005	790
Ilyanassa_obsoleta	4910	800
Aedes_albopictus	153	821
Passiflora_antioquiensis	1467	900
Glycine_max	1223	903
Oryctolagus_cuniculus_	3094	913
Bufo_bufo	5772	940
Bufo_americanus	4707	945
Vicia_benghalensis	2983	950
Rana_pipiens	6487	950
Hyla_versicolor	9536	950
Linum_usitatissimum_	660	980
Cucumis_melo	709	1000
Aedes_flavopictus	1301	1023
Lagenaria_vulgaris	685	1050
Matthiola_incana	1467	1100
Bufo_fowleri	5113	1100
Beta_vulgaris	896	1150
Rana_esculenta	6040	1165
Taxus_baccata	9526	1250
Vicia_villosa	2049	1275
Vigna_radiata	587	1500
Nicotiana_glutinosa	3032	1600
Bellevalia_romana	8293	1600
Oenothera_fructosa	1418	1700
Nicotiana_tabaccum_	4499	1722
Luffa_cylindrica	1125	1800
Vicia_sativa	2023	1888
Ferula_communis	1575	1946
Phaseolus_coccineus	766	2000
Plethodon_glutinosis	27290	2000
Plethodon_elongatus	30166	2000
Bombina_variegata	9474	2050
Juniperus_chinesis_pyramidalis	9562	2050
Plethodon_cinereus	21716	2060
Aquilegia_alpina	538	2300
Nicotiana_tomentosiformis	2753	2320
Tradescantia_paludosa	30081	2400
Neoceratodus_forsteri_	67730	2400
Rhycotriton_olympicus	59365	2430
Plethodon_dunni	40489	2440
Plethodon_vehiculum	37100	2630

Momordica_charantia	2005	2750
Nicotiana_sylvestris	2617	2800
Hordeum_bulbosum	5364	2870
Secale_cereale	8944	2875
Oncorhynchus_mykiss	2528	3030
Vicia_narbonensis	7231	3130
Trillium_grandiflorum	44988	3150
Osmerus_esperlanus	465	3305
Helianthus_annuus	3345	3350
Sprattus_sprattus	518	3375
Nicotiana_rustica	6318	3375
Nicotiana_otophora	3218	3400
Pisum_sativum	4587	3400
Ambystoma_opacum	27133	3500
Hordeum_procerum	5350	3560
Pseudotsuga_douglasii	14196	3600
Oedipina_uniformis	52274	3720
Raphanus_sativus	538	3800
Pseudotriton_ruber_schenki	24487	3900
Ambystoma_talpoideum	29328	3950
Triturus_cristatus	22977	4100
Pseudocalanus_acuspes	3589	4128
Hordeum_vulgare	5347	4200
Necturus_maculosus_	87192	4275
Cucumis_sativus	892	4288
Desmognathus_fuscus	16126	4300
Plethodon_jordani	28329	4300
Tradescantia_virginiana	38387	4300
Ambystoma_mexicanum	33479	4500
Vicia_faba	12791	4750
Hordeum_parodii	5350	4830
Ambystoma_tigrinum	32099	4870
Bellevalia_dubia	11736	4900
Cucurbita_pepo	856	5050
Pinus_sylvestris	23066	5350
Triticum_aestivum	17250	5425
Notophthalmus_viridescens_	39378	5450
Triturus_carnifex	25606	5460
Nicotiana_paniculata	3276	6150
Zea_mays	2894	6425
Calanus_pacificus	6660	6845
Picea_albertiana_	17604	6950
Acartia_hudsonica	958	7056
Salmo_salar	2660	7085
Calanus_finmarchicus	6347	7650
Scilla_peruviana	19047	7750
Phaseolus_vulgaris_	636	7800
Allium_cepa_	16470	7874
Brassica_rapa_	782	9025
Picea_abies_	20440	9650

Salmo_truta	2608	10785
Calanus_hyperboreas	12186	10815
Salvelinus_fontinalis	2768	11865
Picea_sitchensis_	18582	12350
Notophthalmus_viridescens	39259	12500
Larix_decidua_	17179	13400
Calanus_glacialis	11227	16750
Amphiuma_means_	83390	19300
Picea_mariana	17340	22167
Picea_rubens	19296	26048
Heterocapsa_triquetra	22000	4872
Scrippsiella_trochoidea	16000	4112
Prorocentrum_micans	240000	4996
Skeletonema_marinoi	65	61
Ditylum_brightwelli	12000	36896
Phaeodactylum_tricornicum	27	88
Ostreococcus_tauri	13	4
Bathycoccus_prasinos	15	12
Micromonas_pusilla	22	5
Emiliania_huxleyi	168	4
Thalassioria_weisflogii	4597	49
Amphidinium_carterae	11000	840
Prorocentrum_minimum	39000	1432
Gymnodinium_sanguineum	68000	30545
Pelagomonas_calceolata	250	3
Plasmodium_sp	23	6
Saccharomyces_cerevisiae	12	150
Leishmania_major	32	63
Trypanosoma_brucei	24	56
Trypanosoma_cruzi	90	110
Eimeria_tenella	52	140
toxoplasma_gondii	63	110
Yarrowia_lipolytica	20	100
Schizosaccharomyces_pombe	13	110
Pneumocystis_carinii	7	1
Kluyveromyces_lactis	11	60
Hansenula_polymorpha	9	55

Supplementary Table 5				
Cold network:			Warm network:	
Name	No. connections		Name	No. connections
pfam00654	860		pfam16174	1346
pfam00443	858		pfam03957	1231
pfam04408	766		pfam15228	1231
pfam00566	711		pfam15929	1197
pfam01266	664		pfam04107	1181
pfam07717	638		pfam15430	1161
pfam00300	634		pfam05479	1130
pfam00850	630		pfam10195	1127
pfam00176	627		pfam05511	1121
pfam00226	625		pfam17067	1117
pfam10294	581		pfam15410	1111
pfam03798	573		pfam02681	1105
pfam00481	556		pfam00231	1087
pfam00849	551		pfam00416	1084
pfam00583	540		pfam00687	1075
pfam07690	530		pfam02800	1073
pfam13640	526		pfam02915	1073
pfam00856	520		pfam03946	1068
pfam00156	501		pfam00044	1063
pfam01926	480		pfam00828	1063
pfam12146	480		pfam10172	1059
pfam00498	476		pfam13631	1057
pfam00106	470		pfam00347	1049
pfam00107	450		pfam03244	1049
pfam12352	441		pfam00467	1048
pfam01490	435		pfam01716	1048
pfam12697	423		pfam06936	1047
pfam01066	422		pfam01775	1046
pfam10250	408		pfam03764	1044
pfam00149	403		pfam05421	1044
pfam01554	394		pfam03143	1043
pfam04258	391		pfam08207	1040
pfam01625	390		pfam00125	1039
pfam02037	389		pfam00338	1038
pfam04427	378		pfam05757	1038
pfam14833	370		pfam01092	1037
pfam01055	365		pfam01280	1036
pfam02824	357		pfam04457	1036
pfam01399	355		pfam10204	1036
pfam02466	355		pfam07096	1034
pfam01636	354		pfam13183	1032
pfam04755	353		pfam05236	1031
pfam00069	350		pfam01201	1030
pfam04055	350		pfam00252	1029
pfam00271	347		pfam00827	1029
pfam00293	344		pfam00164	1028
pfam00650	343		pfam02201	1028

pfam01529	343		pfam00032	1027
pfam00899	339		pfam00466	1026
pfam00995	339		pfam16906	1026
pfam00248	338		pfam07123	1023
pfam00628	334		pfam04725	1020
pfam03446	328		pfam01282	1019
pfam01751	325		pfam00410	1018
pfam01798	321		pfam03947	1016
pfam00587	317		pfam16205	1016
pfam01040	316		pfam00238	1015
pfam13460	310		pfam00297	1015
pfam06071	309		pfam01158	1015
pfam04178	308		pfam00281	1014
pfam00892	307		pfam02100	1014
pfam13649	305		pfam03297	1014
pfam07731	302		pfam11902	1013
pfam02852	300		pfam01777	1012
pfam00027	296		pfam01157	1011
pfam02358	294		pfam01251	1011
pfam09430	292		pfam01776	1011
pfam00933	291		pfam08642	1011
pfam02516	289		pfam17144	1011
pfam04117	289		pfam00181	1010
pfam00364	286		pfam03501	1008
pfam13489	284		pfam01015	1006
pfam01472	276		pfam01198	1006
pfam01504	274		pfam01090	1005
pfam02847	272		pfam01294	1005
pfam01182	270		pfam00935	1004
pfam01569	270		pfam17135	1003
pfam04511	269		pfam01655	1001
pfam01408	267		pfam03144	1001
pfam00462	266		pfam01248	1000
pfam07534	266		pfam00833	999
pfam00454	260		pfam00162	997
pfam00795	259		pfam00333	996
pfam05577	256		pfam00411	996
pfam01344	253		pfam01778	996
pfam01612	248		pfam04758	995
pfam10436	248		pfam03939	994
pfam01553	247		pfam00177	992
pfam06966	247		pfam00163	990
pfam13621	244		pfam00101	989
pfam14392	244		pfam00504	989
pfam04042	240		pfam00831	989
pfam04577	238		pfam00009	988
pfam13410	235		pfam00485	988
pfam00326	234		pfam01247	986
pfam00557	234		pfam00573	985
pfam05193	229		pfam01246	985

pfam03982	226		pfam01159	982
pfam13086	225		pfam00380	978
pfam00397	220		pfam05151	978

Supplementary Table 6:

Primers for the V4 region of:

16S[2]
FW(515F): GTGCCAGCMGCCGCGGTAA
RV(806R): GGACTACNVGGGTWTCTAAT

18S[12]
FW(565F): CCAGCASCYGCGGTAATTCC
RV(948R): ACTTTCGTTCTTGATYRA

Supplementary References:

1. Boucher, N. *et al.*, Flow cytometric determination of phytoplankton DNA in cultures and oceanic populations. Marine Ecology Progress Series, **71**(1):75–84 (1991).

2. Caporaso *et al.*, Global patterns of 16S rRNA diversity at a depth of millions of sequences per sample. Proc Natl Acad Sci USA 108, 4516–4522 (2011).

3. Carlton, J. M. *et al.*, Malaria parasites: comparative genomics, evolution and molecular biology (2013).

4. Godhe, A. *et al.*, Quantification of diatom and dinoflagellate biomasses in coastal marine seawater samples by real-time PCR. Applied and Environmental Microbiology, 74(23):7174–82 (2008).

5. Hauser, P. M. *et al.*, Comparative Genomics Suggests that the Fungal Pathogen Pneumocystis Is an Obligate Parasite Scavenging Amino Acids from Its Host's Lungs. PLoS ONE, **5**(12):e15152 (2010).

6. Moreau, H. *et al.*, Gene functionalities and genome structure in Bathycoccus prasinos reflect cellular specializations at the base of the green lineage. Genome Biology, 13(8):R74 (2012).

7. NCBI Resource Coordinators, Database resources of the National Center for Biotechnology Information. Nucleic Acids Research, 44(D1):D7-19 (2016).

8. Nordberg, H. *et al.*, The genome portal of the Department of Energy Joint Genome Institute: 2014 updates. Nucleic Acids Research, **42**(D1):D26–D31 (2014).

9. Oliver, M. J. *et al.*, The mode and tempo of genome size evolution in eukaryotes. Genome Research, 17(5):594–601 (2007).

10. Prokopowich, C. D. *et al.*, The correlation between rDNA copy number and genome size in eukaryotes. Genome, **46**(1):48–50 (2003).

11. Rödström, E. M., (2017). Skeletonema marinoi. http://cemeb.science.gu.se/research/target-species-imago+/skeletonema-marinoi

12. Stoeck *et al.*, Multiple marker parallel tag environmental DNA sequencing reveals a highly complex eukaryotic community in marine anoxic water. Mol Ecol 19 Suppl 1:21–31 (2010).

13. Torres-Machorro, A. L. *et al.*, Ribosomal RNA genes in eukaryotic microorganisms: witnesses of phylogeny? FEMS Microbiology Reviews, **34**(1):59–86 (2010).
