# Supplementary material for: The biogeographic differentiation of algal microbiomes in the upper ocean from pole to pole
Source: Nat Commun. 2021 Sep 16;12:5483. doi: 10.1038/s41467-021-25646-9 (PMC8446083; doi:10.1038/s41467-021-25646-9)
Supplement: Supplementary file 3 — Reporting summary [file 41467_2021_25646_MOESM3_ESM.pdf]

nature portfolio

Corresponding author(s): Thomas Mock
Last updated by author(s): Aug 3, 2021

Reporting Summary

Nature Portfolio wishes to improve the reproducibility of the work that we publish. This form provides structure for consistency and transparency in reporting. For further information on Nature Portfolio policies, see our Editorial Policies and the Editorial Policy Checklist.

Statistics

For all statistical analyses, confirm that the following items are present in the figure legend, table legend, main text, or Methods section.

n/a	Confirmed	
☐	☒	The exact sample size (*n*) for each experimental group/condition, given as a discrete number and unit of measurement
☐	☒	A statement on whether measurements were taken from distinct samples or whether the same sample was measured repeatedly
☐	☒	The statistical test(s) used AND whether they are one- or two-sided<br>*Only common tests should be described solely by name; describe more complex techniques in the Methods section.*
☐	☒	A description of all covariates tested
☐	☒	A description of any assumptions or corrections, such as tests of normality and adjustment for multiple comparisons
☐	☒	A full description of the statistical parameters including central tendency (e.g. means) or other basic estimates (e.g. regression coefficient) AND variation (e.g. standard deviation) or associated estimates of uncertainty (e.g. confidence intervals)
☐	☒	For null hypothesis testing, the test statistic (e.g. *F*, *t*, *r*) with confidence intervals, effect sizes, degrees of freedom and *P* value noted<br>*Give P values as exact values whenever suitable.*
☒	☐	For Bayesian analysis, information on the choice of priors and Markov chain Monte Carlo settings
☐	☒	For hierarchical and complex designs, identification of the appropriate level for tests and full reporting of outcomes
☐	☒	Estimates of effect sizes (e.g. Cohen's *d*, Pearson's *r*), indicating how they were calculated

Our web collection on statistics for biologists contains articles on many of the points above.

Software and code

Policy information about availability of computer code

Data collection	No software was used for data collection.
Data analysis	rrnDB database version (5.3)<br>Metagenome Annotation Pipeline (MAP) (v4.15.2)<br>HMMER (3.1b2)<br>Pfam (v30)<br>SILVA database (SSUREF 115)<br>CD-HIT (4.6.1)<br>ClustalW (2.1)<br>TrimAL (1.1)<br>RaxML (8.0.20)<br>NCBI taxtastic tool (0.8.4)<br>HMMER3 (3.1b1)<br>pplacer (1.1)<br>megan (5.10.3)<br>JGI pipeline iTagger v2.1 16S classification pipeline<br>Duk<br>cutadapt<br>FLASH<br>BBMap<br>Bbmerge<br>BBNorm

Rnnotator
MEGAHIT
NCBI genome database (October 2015)
USEARCH (v8.1.1861_i86linux32)

The custom code has been uploaded to GitHub (https://github.com/SeaOfChange/SOC/blob/master/get_ref_seqs.pl). The species of interest were selected from the SILVA database, classified with NCBI taxa IDs and a sequence information file produced that describes each of the algae sequences by their sequence ID and NCBI species ID. Taxonomy from the NCBI database, eukaryote sequences from the SILVA database and a list of algal taxa including outgroups were used as input for the script.

For manuscripts utilizing custom algorithms or software that are central to the research but not yet described in published literature, software must be made available to editors and reviewers. We strongly encourage code deposition in a community repository (e.g. GitHub). See the Nature Portfolio guidelines for submitting code & software for further information.

Data

Policy information about availability of data

All manuscripts must include a data availability statement. This statement should provide the following information, where applicable:
- Accession codes, unique identifiers, or web links for publicly available datasets
- A description of any restrictions on data availability
- For clinical datasets or third party data, please ensure that the statement adheres to our policy

Supplementary information is available in the online version of the paper. Reprints and permission information is available online at www.nature.com/reprints. Correspondence and requests for materials should be addressed to TM.

iTAG rDNA Data:
https://opendata.earlham.ac.uk/opendata/data/ocean_metagenomic/

Eukaryotic metatranscriptome data:
https://genome.jgi.doe.gov/portal/SeaofArctiOcean/SeaofArctiOcean.info.html
(DOI:10.25585/1488054)

Field-specific reporting

Please select the one below that is the best fit for your research. If you are not sure, read the appropriate sections before making your selection.

☐ Life sciences ☐ Behavioural & social sciences ☒ Ecological, evolutionary & environmental sciences

For a reference copy of the document with all sections, see nature.com/documents/nr-reporting-summary-flat.pdf

Ecological, evolutionary & environmental sciences study design

All studies must disclose on these points even when the disclosure is negative.

Study description: Samples for metatranscriptome (n=79) and rDNA (n=57 (16s), n=54 (18s)) sequencing were collected from pole to pole at the chlorophyll a maximum layer in the surface ocean.

Research sample: Microbial phytoplankton (both eukaryotic and prokaryotic) from the surface ocean over a global latitudinal gradient.

Sampling strategy: Samples for meta-omics and rDNA data were chosen to cover large-scale latitudinal gradients including polar oceans. The eukaryotic metatranscriptomes are from pole-to-pole. The metatranscriptome and rDNA samples were sequenced according to JGI protocols.

Data collection: Water samples from the Arctic Ocean and South Atlantic Ocean expeditions (ARK-XXVII/1 (PS80) and ANT-XXIX/1 (PS81)) were collected using 12L Niskin bottles (Rosette sampler with an attached Sonde (CTD, conductivity, temperature, depth) either at the chlorophyll maximum (10-110m) and/or upper of the ocean (0-10m). As soon as the rosette sampler was back on board, water samples were immediately transferred into plastic containers and transported to the laboratory. All samples were accompanied by measurements on salinity, temperature, sampling depth and silicate, nitrate, phosphate concentration. Water samples were pre-filtered with a 100 µm mesh to remove larger organisms and subsequently filtered onto 1.2 µm polycarbonate filters (Isopore membrane, Millipore, MA, USA). All filters were snap frozen in liquid nitrogen and stored at -80°C until further analysis. These samples were collected by Dr. Katrin Schmidt of the University of East Anglia

Water samples from the North Atlantic Ocean cruise (Stratiphyt-II) were also taken with 12L Niskin bottles attached to a Rosette sampler with a Sonde. However, these samples were filtered onto 0.2 µm polycarbonate filters (Isopore membrane, Millipore, MA, USA) without pre-filtration but snap frozen in liquid nitrogen and stored at -80°C as the other samples. These samples were collected by Dr. Willem van de Poll of the University of Groningen, Netherlands and Dr. Klaas Timmermans of the Royal Netherlands Institute for Sea Research

Water samples from the Southern Ocean cruise (ANT-XXXII/2 (PS103)) were taken with 12 L Niskin bottles attached to an SBE911plus CTD system equipped with 24 Niskin samplers. These samples were filtered onto 1.2 µm polycarbonate membrane filters (Merck

	Millipore, Germany) in a container cooled to 4 °C and snap frozen in liquid nitrogen and stored at -80°C as the other samples. These samples were collected by Dr. Allison Fong of the Alfred-Wegener Institute for Polar and Marine Research, Bremerhaven, Germany.
Timing and spatial scale	ARK-XXVII/1 (PS80) - 17th June to 9th July 2012; Stratiphyt-II - 1st April to 30th May 2011; ANT-XXIX/1 (PS81) - 1st to 24th November 2012 and ANT-XXXII/2 (PS103) - 16th December 2016 to 3rd February 2017 and covers a transect of the Atlantic Ocean from Greenland to the Weddell Sea (71.36°S to 79.09°N).
Data exclusions	No data were excluded from the analyses.
Reproducibility	This is based on long-term field work over a global latitudinal gradient.
Randomization	Not relevant as the sampling was based on targeted geographic locations
Blinding	Not relevant as the sampling was based on targeted geographic locations

Did the study involve field work? ☒ Yes ☐ No

Field work, collection and transport

Field conditions	Field work was based on the project "Sea of Change: Eukaryotic Phytoplankton Communities in the Arctic Ocean" (DOI: 10.25585/1488054) hosted at the Joint Genome Institute (USA). This data set consists of sequence data from 4 separate cruises: ARKXXVII/1 (PS80) - 17th June to 9th July 2012; Stratiphyt-II - 1st April to 30th May 2011; ANT-XXIX/1 (PS81) - 1st to 24th November 2012 and ANT-XXXII/2 (PS103) - 20th December 2016 to 26th January 2017 and covers a transect of the Atlantic Ocean from Greenland to the Weddell Sea (71.36°S to 79.09°N). For more details see: PhD thesis of Schmidt, K. (2017). Thermal adaptation of Thalassiosira pseudonana using experimental evolution approaches (University of East Anglia) and https://www.pangaea.de/expeditions/cr.php/ Polarstern (Alfred-Wegener Institute, Bremerhaven, Germany).
Location	https://www.pangaea.de/expeditions/cr.php/Polarstern
Access & import/export	Samples were taken with the icebreaker RV Polarstern.
Disturbance	Sampling the surface ocean only causes minimal disturbance of the ecosystem.

Reporting for specific materials, systems and methods

We require information from authors about some types of materials, experimental systems and methods used in many studies. Here, indicate whether each material, system or method listed is relevant to your study. If you are not sure if a list item applies to your research, read the appropriate section before selecting a response.

Materials & experimental systems

n/a	Involved in the study
☒	☐ Antibodies
☒	☐ Eukaryotic cell lines
☒	☐ Palaeontology and archaeology
☒	☐ Animals and other organisms
☒	☐ Human research participants
☒	☐ Clinical data
☒	☐ Dual use research of concern

Methods

n/a	Involved in the study
☒	☐ ChIP-seq
☒	☐ Flow cytometry
☒	☐ MRI-based neuroimaging
